# IBR5 Regulates Leaf Serrations Development via Modulation of the Expression of *PIN1*

**DOI:** 10.3390/ijms20184429

**Published:** 2019-09-09

**Authors:** Xiuzhen Kong, Guoqiang Huang, Yali Xiong, Chunyan Zhao, Jun Wang, Xiaoyun Song, Jitender Giri, Kaijing Zuo

**Affiliations:** 1 Plant Biotechnology Research Center, School of Agriculture and Life Sciences, Shanghai Jiao Tong University, Shanghai 200240, China (X.K.) (Y.X.) (C.Z.) (J.W.) (X.S.); 2 State Key Laboratory of Hybrid Rice, School of Life Sciences and Biotechnology, Shanghai Jiao Tong University, Shanghai 200240, China; 3 National Institute of Plant Genome Research, New Delhi 110067, India

**Keywords:** *Arabidopsis*, leaf serrations, IBR5, auxin maxima, PIN1

## Abstract

Biodiversity in plant shape is mainly attributable to the diversity of leaf shape, which is largely determined by the transient morphogenetic activity of the leaf margin that creates leaf serrations. However, the precise mechanism underlying the establishment of this morphogenetic capacity remains poorly understood. We report here that INDOLE-3-BUTYRIC ACID RESPONSE 5 (IBR5), a dual-specificity phosphatase, is a key component of leaf-serration regulatory machinery. Loss-of-function mutants of *IBR5* exhibited pronounced serrations due to increased cell area. IBR5 was localized in the nucleus of leaf epidermis and petiole cells. Introducing a C129S mutation within the highly conserved VxVHCx_2_GxSRSx_5_AYLM motif of IBR5 rendered it unable to rescue the leaf-serration defects of the *ibr5-3* mutant. In addition, auxin reporters revealed that the distribution of auxin maxima was expanded ectopically in *ibr5-3*. Furthermore, we found that the distribution of PIN1 on the plasma membrane of the epidermal and cells around the leaf vein was compromised in *ibr5-3*. We concluded that IBR5 is essential for the establishment of PIN-FORMED 1 (PIN1)-directed auxin maxima at the tips of leaf serration, which is vital for the elaborated regulation during its formation.

## 1. Introduction

Leaf serrations determine the morphology of leaf shape, which mainly contributes to the biodiversity of plant shape [1,2,3]. On this basis, leaves with slight serrations are classified as simple leaf, while leaves with strong dissections are termed compound leaves [4]. Hence, the development of leaf serration is an intriguing process, attracting much attention from many scientists.

Recent molecular studies demonstrated that the formation of leaf serration is a sophisticated process that is fine-tuned by various environmental and developmental factors, such as light, phytohormone, and temperature [3,5,6,7,8]. Among these factors, auxin is critical in determining the morphology of leaf serrations [9]. As an essential hormone, auxin is involved in almost every biological activity, ranging from gravitropism [10] to the abiotic stress response [11], and from cell expansion [12] to the formation of leaf margin [9,13]. The morphogenesis of leaf serrations depends on the guidance of auxin maxima at their tips [9], and that is regulated by many factors, such as four auxin biosynthesis proteins, YUCCA1/2/4/6 (YUC1/2/4/6), Indole-3-Acetic Acid Carboxyl Methyltransferase (IAMT1), auxin influx carrier AUXIN-RESISTANT 1 (AUX1), and auxin efflux protein PIN-FORMED 1 (PIN1) [14,15,16,17,18,19,20,21].

In addition to the reported factors in auxin signaling pathways, CUP-SHAPED COTYLEDON2 (CUC2) plays vital roles in the generation of auxin maxima through promotion of the polar distribution of PIN1 [13]. Conversely, auxin also represses the expression of *CUC2* during the morphogenesis of leaf serrations [9,13]. Moreover, auxin and *ASYMMETRIC LEAVES1* (*AS1*) signaling pathways converge to suppress the expression of the KNOTTED1-like homeobox (KNOX) gene *BREVIPEDICELLUS* (*BP*), which plays a key role in generating the auxin maxima at the tips of leaf serrations [14]. Despite the known importance of auxin maxima in determining the formation of leaf serrations, the factors affecting the establishment of auxin maxima in this process remain mostly unknown.

Mitogen-activated protein kinases (MAPKs) form a highly conserved family of proteins that are dephosphorylated by MAPKs phosphatases (MKPs) in a large number of physiological processes [22]. *IBR5* encodes one of the five dual-specificity phosphatases (MKP1, MKP2, DUAL-SPECIFICITY PROTEIN TYROSINE PHOSPHATASE 1 (DsPTP1), PROPYZAMIDE HYPERSENSITIVE 1 (PHS1), and INDOLE-3-BUTYRIC ACID RESPONSE 5 (IBR5) and that all of them contain a highly conserved dual-specificity motif VxVHCx_2_GxSRSx_5_AYLM in *Arabidopsis* [22,23]. MKP2 and DsPTP1 have been reported to possess phosphatase activity against MPK3/6 and MPK4, respectively [24,25]. Dephosphorylation of MPK12 by IBR5 was confirmed in auxin signaling pathways in the root [26]. The expression of *IBR5* has been detected in almost all tissues throughout developmental stages, including roots, petals, sepals, anther filaments, carpels of flowers, green siliques, and leaf serrations [23], and loss-of-function mutants of *IBR5* displayed pleiotropic phenotypes, such as longer roots, shorter hypocotyls, smaller petals, and pronounced serrations [23,26,27]. However, the molecular mechanism of IBR5-mediated leaf serration formation remains unknown.

In this study, we report the functional analysis of *IBR5* in the development of leaf serrations. IBR5 was constitutively expressed and localized in the nucleus of leaf epidermal cells. Loss-of-function mutants of *IBR5* exhibited pronounced leaf serrations due to enhanced cell expansion but not normal cell proliferation. Moreover, IBR5 was necessary for the establishment of auxin maxima that is restricted at the tips of leaf serrations. The abundance of PIN1 on the plasma membrane of the epidermal cells and cells around the leaf vein were compromised in *ibr5-3*. Also, IBR5_C129S_ lost the ability to rescue the defective leaf serrations of *ibr5-3*. Our results demonstrated that IBR5 is a critical factor in the regulation of leaf serration development by altering auxin maxima distribution.

## 2. Results

### 2.1. Identification and Phenotypic Characterization of IBR5 Loss-of-Function Mutants

**Figure 1:**
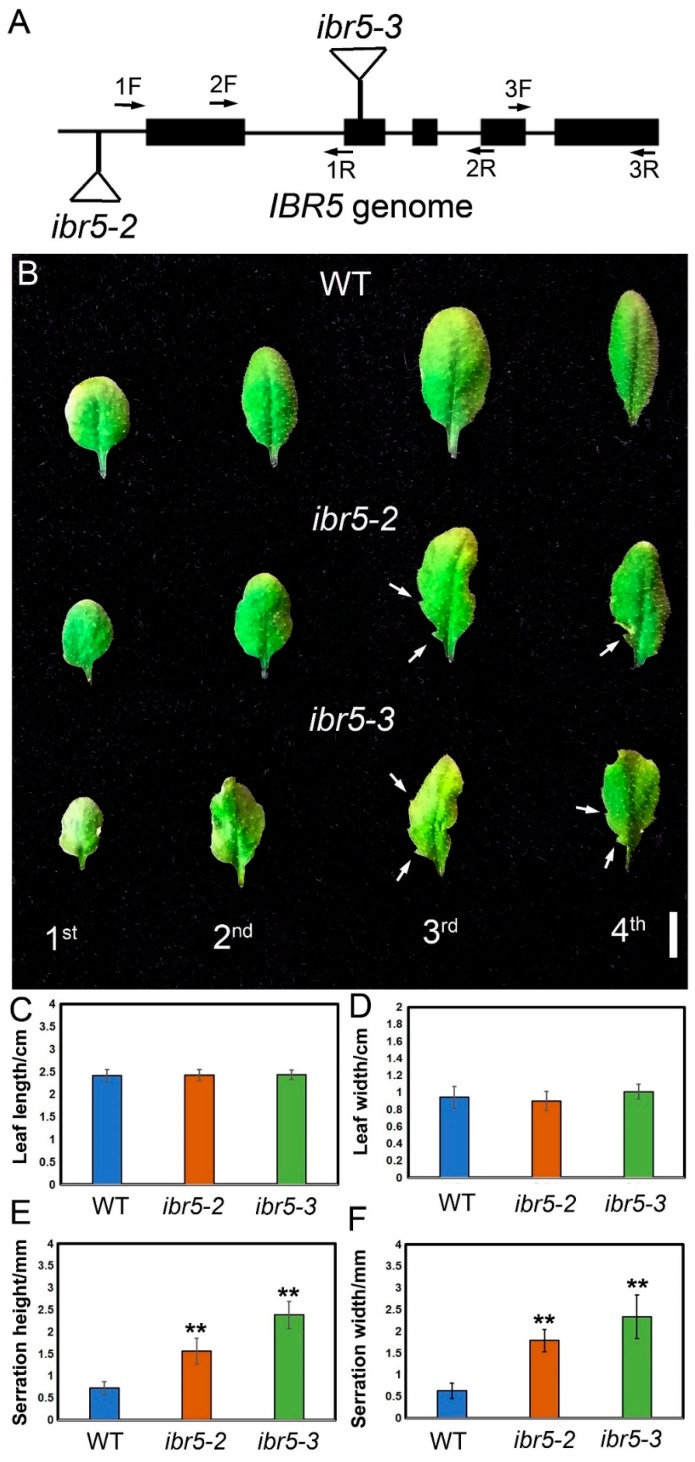
*ibr5-3* mutants showing defective leaf shape. (**A**) The schematic illustration of the T-DNA insertion mutants for *IBR5* used in this study [23,26,27]. (**B**) Phenotypic characterization of WT, *ibr5-2* and *ibr5-3*. The white arrows indicate the leaf serrations. Bar, 1 cm. (**C**) The length analysis of the third leaf in WT, *ibr5-2*, and *ibr5-3*. Error bars indicate ±SD; 12 leaves from 12 individual plants were analyzed. (**D**) The width analysis of the third leaf in WT, *ibr5-2*, and *ibr5-3*. Error bars are ±SD; 12 leaves from 12 individual plants were analyzed. (**E**) The maximum height analysis of the most proximal serrations of the third leaf in WT, *ibr5-2*, and *ibr5-3*. Error bars are ± SD; 11 leaves from 11 individual plants were analyzed. Two asterisks mean significant differences (*p* < 0.01 from Student’s *t*-test). (**F**) The maximum width analysis of the most proximal serrations of the third leaf in WT, *ibr5-2*, and *ibr5-3*. Error bars are ±SD; 11 leaves from 11 individual plants were analyzed. Two asterisks mean significant differences (*p* < 0.01 from Student’s *t*-test).

The development of leaf serrations is an intriguing process, which has triggered our interests. Using a reverse genetic approach to understand the molecular mechanism underlying the development of leaf serrations, we analyzed two T-DNA insertion mutants exhibiting defective leaf serration development. We found that the T-DNA fragments of the two mutants were inserted into the sequence of the *IBR5* locus. In agreement with a previous report [23,26,27], one T-DNA fragment was inserted at the 5′ untranslated region at 161 bp upstream of the ATG and the other was inserted in the and second exon of the *IBR5* genomic sequence. These two mutants were termed *ibr5-2* (SALK_032185) and *ibr5-3* (SALK_039359C), respectively (Figure 1A) [23,26,27]. The upstream and downstream sequences of the inserted sites were intact in these two mutants as revealed by PCR analysis. To verify whether the T-DNA insertion disrupted the expression of *IBR5*, we conducted qRT-PCR analysis using total RNAs isolated from the leaves of WT and *ibr5* mutants with three paired primers designed to amplify three different regions of the transcript. Decreased expression of *IBR5* was detected in *ibr5-2*, which suggested this mutant was a weak allele with the full-length transcript at a reduced level (Supplementary Figure S1A–C). However, only the portion of the transcript that was upstream of the T-DNA insertion site could be detected in *ibr5-3*, implying that this mutant produced a truncated IBR5 with the first 100 amino acids. Hence, *ibr5-3* was used for further analysis unless stated otherwise.

Homozygous *ibr5* mutants showed developmental defects in leaf serrations as compared to the wild type (WT) when grown in soil (Figure 1B and Supplementary Figure S2), but the length and width of the first to fourth leaves were normal in these mutants (Figure 1C,D). However, the shape of the leaf margin in *ibr5* mutants was extremely irregular due to the increased height and width of the leaf serrations (Figure 1E,F and Supplementary Figure S3A–F). To investigate the underlying reasons for these defects, the cell size and number were monitored in the most proximal leaf serrations of the third leaves in WT and *ibr5-3*. We observed a larger cell size and normal cell number in *ibr5-3* (Supplementary Figure S4A–D). Here, we conclude that the pronounced serrations of *ibr5-3* were due to a larger cell size, which suggested that IBR5 is involved in cell expansion during the formation of leaf serrations.

### 2.2. IBR5 but Not IBR5_C129S_ Rescues the Leaf-Serration Defects of ibr5-3

**Figure 2:**
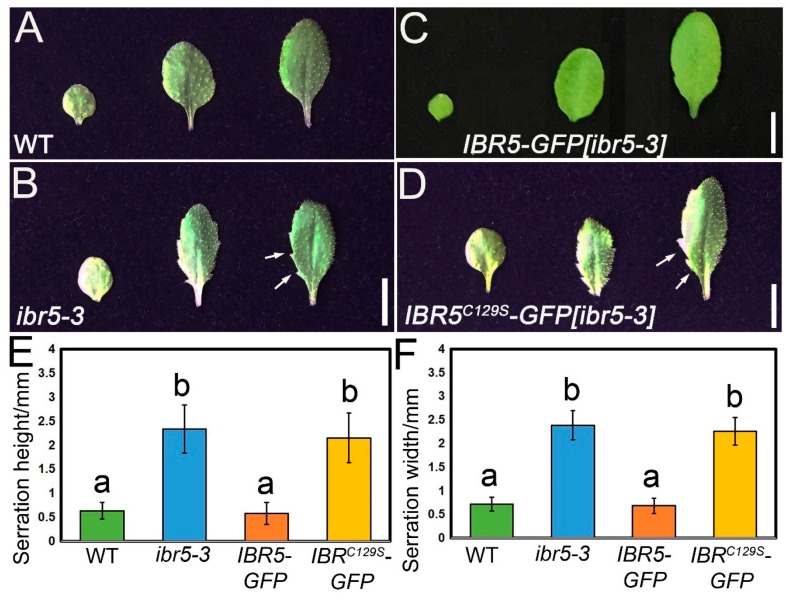
The functionality of IBR5 relies on its dual-specificity phosphatase activity. (**A**) Representative images of the first, second, and third leaves in the WT. Bar, 5 mm. (**B**) Representative images of the first, second, and third leaves in the WT. Bar, 5 mm. (**C**) Representative images of the first, second, and third leaves in *IBR5_pro_::IBR5 _genomic fragment_–GFP* rescued lines. Bar, 5 mm. (**D**) Representative images of the first, second, and third leaves in *IBR5_pro_::IBR5 _genomic fragment C126S_–GFP* rescued lines. Bar, 5 mm. (**E**) The maximum height analysis of the most proximal serrations of the third leaf in WT, *ibr5-3*, and rescued lines. Error bars are ± SD, with 13 leaves analyzed. Different characters mean significant differences (*p* < 0.01 from Student’s *t*-test). (**F**) The maximum width analysis of the most proximal serrations of the third leaf in the WT and *ibr5-3*. Error bars are ± SD, with 11 leaves analyzed. Different characters mean significant differences (*p* < 0.01 from Student’s *t*-test).

Previous studies showed that *ibr5* mutants exhibit pleiotropic defects, such as slight dwarfing, fewer lateral roots, and smaller petals [23,28,29]. Here, we showed that a pronounced leaf serration phenotype is associated with this mutant (Figure 1B,E,F). *IBR5* was expressed in almost all tissues analyzed by microarrays [30]. To further validate its predicted expression pattern, we introduced an *IBR5* genomic fragment fused with an in-frame GFP (*IBR5-GFP*) into the *ibr5-3* background. The *IBR5* genomic fragment fully rescued the defects of leaf serrations in *ibr5-3* (Figure 2A–C,E,F), demonstrating that GFP translational fusion did not affect the function of IBR5. Therefore, complementary lines could be employed to study IBR5 subcellular localization in different tissues.

Given that IBR5 is a dual-specificity phosphatase, we wonder whether its regulation on the development of leaf serrations relied on its catalytic activity. Hence, we introduced a point mutation into the sequence of *IBR5* and in which the conserved VxVHCx_2_GxSRSx_5_AYLM motif was changed to VxVHSx_2_GxSRSx_5_AYLM (IBR5_C129S_). This mutation has been shown to abolish the ability of IBR5 to trigger the dephosphorylation process with a nucleophilic attack on the phosphorus atom of the phosphotyrosine or phosphothreonine substrate [25,31,32]. We introduced an IBR5_C129S_–GFP fusion fragment into *ibr5-3* and found that the exogenous IBR5_C129S_–GFP failed to compensate for the defects of its leaf serrations (Figure 2A,B,D–F), suggesting that IBR5-mediated development of leaf serrations is dependent on its phosphatase activity.

### 2.3. IBR5 Is Expressed and Localized in the Nucleus of Cells Forming the Leaf

**Figure 3:**
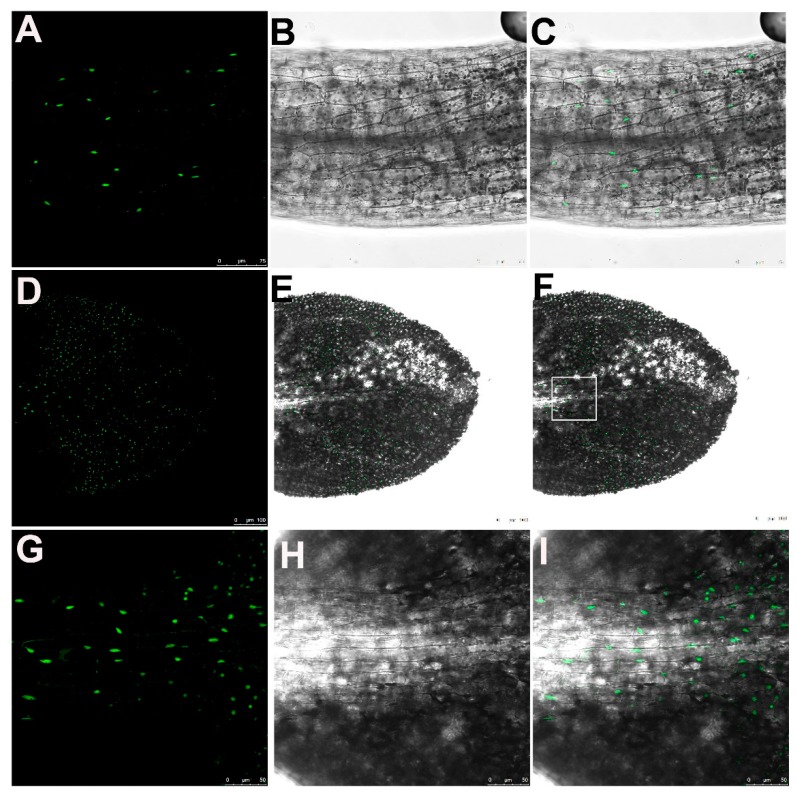
Expression pattern and subcellular localization of IBR5. (**A**–**C**) Representative images of IBR5 expressed and localized in the nucleus of petiole cells. Bars, 75 µm. (**D**–**F**) Representative images of IBR5 expressed and localized in the nucleus of epidermal cells of 2-day-old leaves. Bars, 100 µm. (**G**–**I**) Representative images of IBR5 expressed and localized in the nucleus in the boxed region in the figure (**F**). Bars, 100 µm.

To better understand the roles of IBR5 in the development of leaf serrations, it is necessary to define its subcellular localization. Recent studies conducted by transient expression in tobacco leaf epidermal cells showed that *IBR5* generated two spliced transcripts *IBR5.1* and *IBR5.3*, which would be translated into two products localized in both the nucleus and cytoplasm or only the nucleus, respectively [27]. As a fast and convenient approach, transient heterozygous expression could not always reveal the real subcellular localization, especially in some tissues, such as petiole and leaf vascular bundles. Therefore, we used stable complementary *Arabidopsis* lines in the *ibr5* mutant background (IBR5–GFP). Confocal imaging showed that clear and intense GFP signals were accumulated in the nucleus of the petiole and cells around the leaf vein (Figure 3). Furthermore, only IBR5.1–GFP was detected in the leaves of fully rescued IBR5–GFP plants (Supplementary Figure S5), which indicates that IBR5.1 but not IBR5.3 functions in leaf serration development.

### 2.4. Auxin Distribution Is Perturbed in ibr5-3

Next, we attempted to investigate the roles of auxin in the development of leaf serrations. Auxin maxima were demonstrated to act as a critical factor for determining the size of leaf serrations [9,13]. Given the enhanced cell expansion in *ibr5* mutants (Supplementary Figure S4A–C), we hypothesized that auxin maxima were perturbed in these mutants.

**Figure 4:**
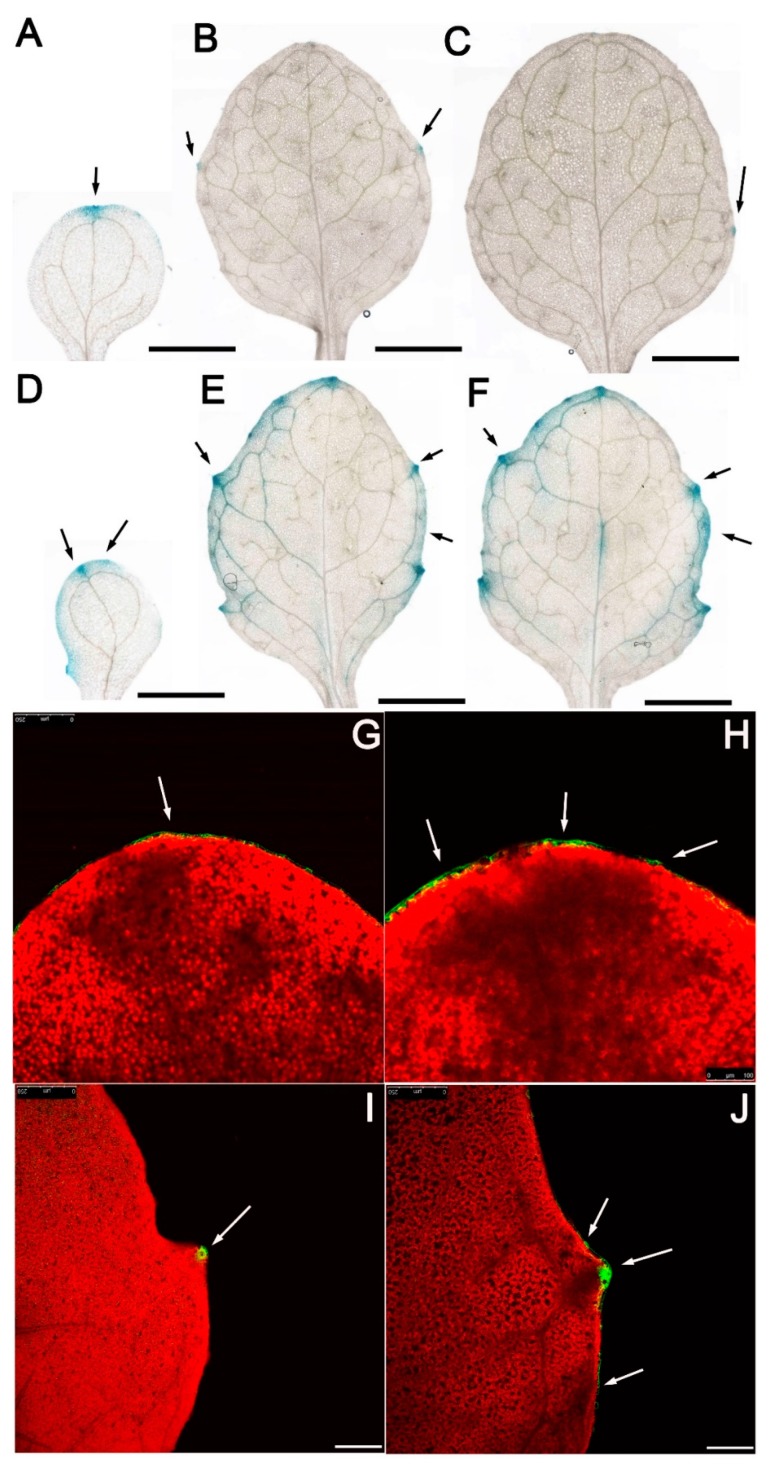
Auxin accumulation monitored by GUS staining and confocal microscopy in *ibr5-3*. (**A**–**C**) Representative images of GUS staining for 10-day-old DR5-GUS transgenic plants in WT. Bars, 5 mm. From left to right: cotyledon, the third true leaf, and the fifth true leaf. The black arrows indicate the auxin maxima sites. (**D**–**F**) The representative images of GUS staining for 10-day-old DR5-GUS transgenic plants in *ibr5-3*. Bars, 5 mm. From left to right: cotyledon, the third true leaf, and the fifth true leaf. The black arrows indicate the auxin maxima sites. (**G**,**H**) Representative images of confocal imaging for the third leaf of 10-day-old DR5-GFP transgenic plants in WT. Bars, 100 µm. The white arrows indicate the auxin maxima sites. (**I**,**J**) Representative images of confocal imaging for the third leaf of 10-day-old DR5-GFP transgenic plants in *ibr5-3*. Bars, 100 µm. The white arrows indicate the auxin maxima sites.

To determine if auxin distribution was perturbed in *ibr5-3*, we crossed an auxin reporter line DR5:GUS [33] into *ibr5-3* and analyzed the GUS signals in vivo. The punctuated GUS signals were spatially restricted at the tips of 10-day-old leaf serrations and leaf apex in the WT (Supplementary Figure S6), consistent with previous work [9]. By contrast, strong and ectopic GUS signals expanded from the leaf apex and the tips of leaf serrations to nearly the whole leaf margin in *ibr5-3* (Supplementary Figure S6), demonstrating that enhanced auxin response and perturbed auxin maxima existed in *ibr5* mutants. To obtain high-quality images for the distributed sites of GUS signals, we analyzed the first, third, and fourth leaves by light transmission microscopy. We found that GUS signals were not only distributed at the leaf apex and the tips of leaf serrations in *ibr5-3* (Figure 4A–C) but expanded to most if not all the sinus and covered the whole leaf margin in these three leaves (Figure 4E,F). To confirm these results, we then introduced another auxin reporter line DR5-GFP into *ibr5-3* and WT plants. Similarly, the third leaves of WT and *ibr5-3* were selected as targets for further analysis. Confocal imaging showed that GFP signals were spatially restricted and accumulated at the leaf apex and the tips of leaf serrations (Figure 4G,I), demonstrating that the auxin maxima were strictly restricted to the tips of the leaf serrations and leaf apex. While, more GFP signals were accumulated and ectopically expanded from the leaf apex and tips of leaf serrations to the adjacent regions in *ibr5-3* (Figure 4H,J), a similar result that was also observed in DR5:GUS lines. Since auxin maxima at the tips of leaf serrations are responsible for determining the formation of leaf serrations [34], the pronounced serrations observed in *ibr5-3* can be explained reasonably by the enhanced auxin response and ectopic auxin maxima (Figure 4D–F). Collectively, we propose that the pronounced serrations were due to the ectopic auxin maxima and more active auxin response in *ibr5-3*.

### 2.5. The Distribution of PIN1 Is Compromised in ibr5-3

We have shown that the auxin response was enhanced in the leaf apex and the tips of leaf serrations. We next investigated why an enhanced auxin response was characterized in *ibr5* mutants. Leaves serve as the source to produce auxin that is necessary for the development of other organs [35,36]. Firstly, we determined the transcriptional level of auxin biosynthetic genes expressed in leaves, *YUC1*, *YUC2*, *YUC4*, and *YUC6* [19,20,28], and our qRT-PCR results showed that the expression levels of these four genes were not affected in the *ibr5* mutant (Supplementary Figure S7). These results suggested that the process of auxin biosynthesis was normal in *ibr5-3*. 

**Figure 5:**
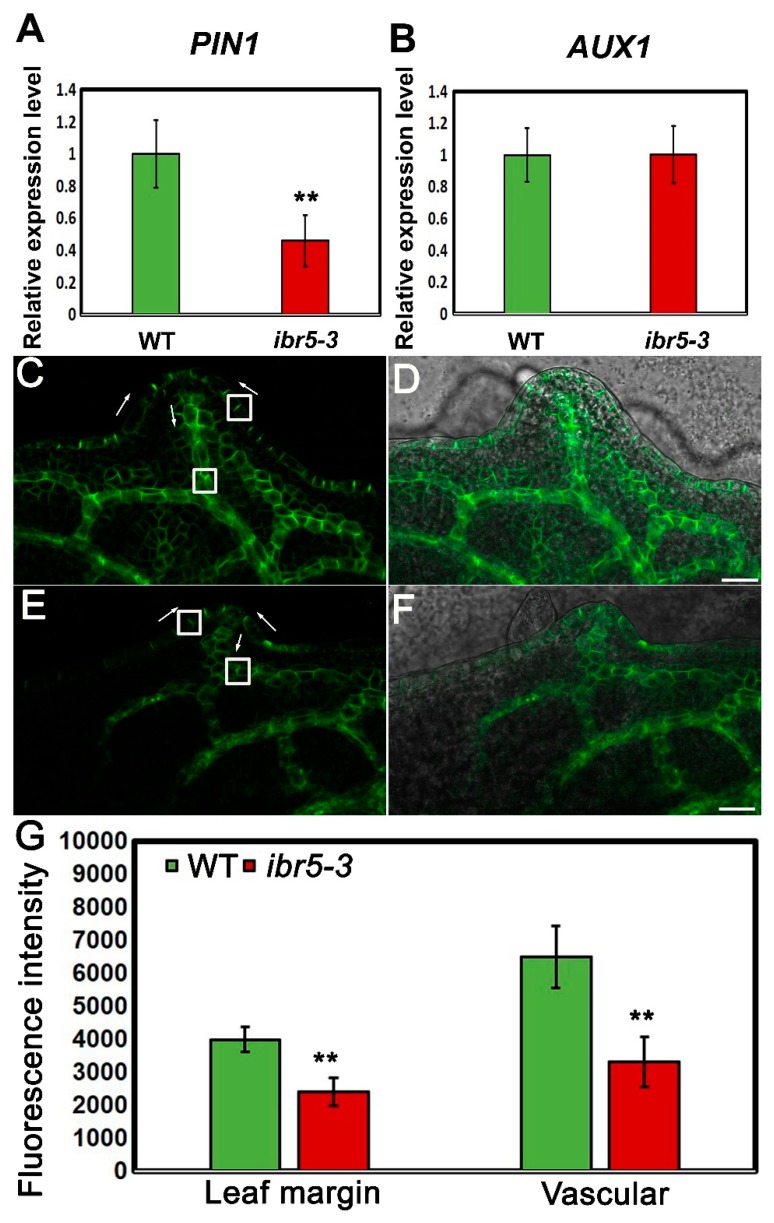
The distribution of PIN1 in the leaf serrations of WT and *ibr5-3*. (**A**) The relative expression level of *PIN1* in WT and *ibr5-3*. Error bars are ± SE, *n* = 3 independent replicates and each with 3 biological replicates analyzed in each assay. (**B**) The relative expression level of *AUX1* in WT and *ibr5-3*. Error bars are ± SE, *n* = 3 independent replicates and each with 3 biological replicates analyzed in each assay. (**C**,**D**) The representative image of PIN1-GFP in the 10-day-old leaf serrations of WT. Bar, 20 µm. The white boxed regions were used for analysis. The white arrows indicate the direction of auxin transport. (**E**,**F**) The representative image of PIN1-GFP in the 10-day-old leaf serrations of *ibr5-3*. Bar, 20 µm. The white boxed regions were used for analysis. The white arrows indicate the direction of auxin transport. (**G**) The quantified analysis of the fluorescence intensity of PIN1-GFP in 10-day-old leaf serrations in WT and *ibr5-3*. Error bars are ± SE, *n* = 3 independent replicates and each with 12 leaves analyzed in each assay. Two asterisks mean significant differences (*p* < 0.01 from Student’s *t*-test).

PIN1 is an auxin efflux carrier and primarily responsible for the polar transport of auxin, and its convergence points mark the auxin maxima guiding the formation of leaf serrations [4,14,17,34]. We wondered whether the expression and localization of PIN1 were affected in *ibr5-3*. qRT-PCR results showed that the transcriptional level of *PIN1* but not *AUX1* was decreased in *ibr5-3* (Figure 5A,B), implying the abundance of PIN1 was related to IBR5. We then employed a PIN1-GFP transgenic line [36] to mark the localization and accumulation of PIN1 and crossed it into *ibr5-3*. GFP signals were distributed asymmetrically on the plasma membrane of epidermal and cells around the leaf vein of leaf serrations in the WT background (Figure 5C,D), which indicated the auxin transport pathway (i.e., from the sinus to the tip of serrations and finally into vascular tissue) as reported [9]. GFP signals were also found to be localized on the plasma membrane of cells around the leaf vein and epidermal cells in *ibr5-3* (Figure 5E,F), demonstrating that the polar localization of PIN1 was not affected and the direction of auxin transport was normal in the absence of *IBR5*. However, the intensity of the GFP signal was much weaker on the plasma membrane of both epidermal cells and cells around the leaf vein in *ibr5-3* versus WT (Figure 5E), indicating that the abundance of PIN1 on the plasma membrane was compromised in *ibr5-3*. Given that PIN1 acts as an auxin efflux carrier, the reduced abundance of PIN1 on the plasma membrane likely led to the reduced rate of auxin transport. Hence, the transport pathway of auxin produced in the leaf was likely to be partially limited, which may cause an enhanced auxin response and auxin maxima expanded from the tips of leaf serration to the adjacent regions in *ibr5-3*.

Here, we conclude that IBR5 is a critical factor in the formation of leaf serrations through modulation of the abundance of PIN1 on the plasma membrane of leaf epidermal and cells around the leaf vein, which is responsible for transporting auxin produced by leaves to other organs. The blockage of this pathway may lead to an enhanced auxin response in the leaf margins and pronounced leaf serrations.

## 3. Discussion

**Figure 6:**
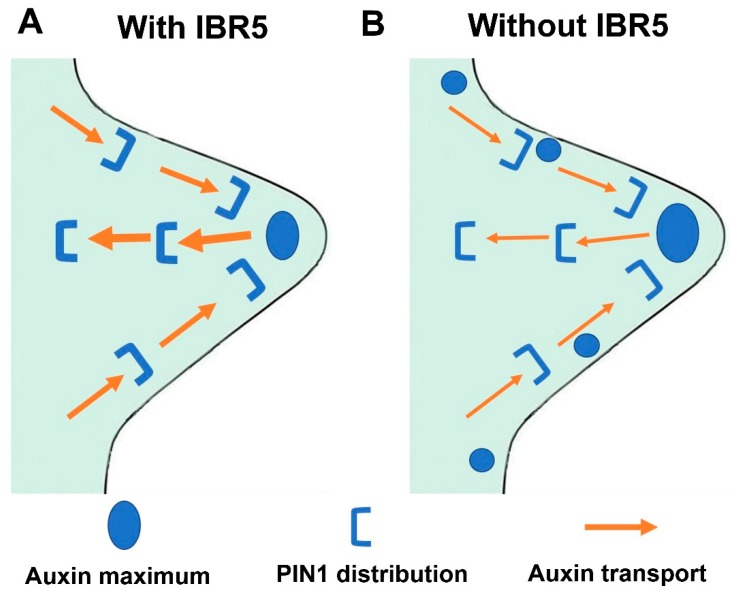
The schematic model for this work. (**A**) With IBR5, auxin maxima were restricted at the tips of leaf serrations. (**B**) Without IBR5, the distribution of PIN1 on the plasma membrane of the epidermal and cells around the leaf vein was compromised, the rate of auxin transport was reduced, and auxin maxima were enlarged and expended from the tip to the adjacent regions.

Plant leaves are essential for light harvest, gas exchange, starch accumulation, and phytohormone production [1,4,36,37,38]. These processes are significantly influenced by the morphology of leaf shape, which is primarily determined by the size of leaf serrations [13]. Studies on the regulatory mechanism underlying the development of leaf serrations have revealed a role for the auxin signaling network in this process [9,13]. Moreover, the expression of *IBR5* was induced by external auxin [29]. Here we demonstrated that IBR5 is a key regulator in the development of leaf serrations through modulation of the accumulation of PIN1 on the plasma membrane of the epidermal and cells around the leaf vein. PIN1 is critical for regulating the rate of auxin transport and restricting the auxin maxima to the tips of leaf serrations (Figure 6). IBR5 was highly expressed in almost every tissue, and consequently, its mutants exhibited pleiotropic defects, such as slight dwarfism, smaller petals, and defective leaf vasculature patterns [23,27,28]. Here, we showed that an enhanced auxin response was characterized in leaf serrations of the *ibr5* mutant (Figure 4). Our studies systematically investigated the molecular mechanisms of IBR5 in the development of leaf serrations. Moreover, we provided mutually verifiable evidence to support our conclusion that excessive and ectopic auxin distribution exists in the leaf serrations of *ibr5-3* mutants.

Auxin is an important hormone that plays a critical role in determining the shape of leaf serrations [9,13]. *ibr5* mutants exhibited defective leaf shapes due to pronounced leaf serrations (Figure 1B), which was similar to the loss-of-function mutants of *AS1* and *AUXIN RESISTANT1* (*AXR1*) caused by the abnormal distribution of auxin maxima [14]. We initially speculated that this phenotype was caused by perturbed auxin maxima as implied by the previous study [9]. Indeed, auxin maxima were expanded ectopically in the tips of leaf serrations in *ibr5-3* (Figure 4B) due to blocked auxin transport caused by the compromised PIN1 abundance on the plasma membrane of the epidermal and cells around the leaf vein of leaf serrations (Figure 4 and Figure 5). This demonstrated once again that PIN1 is a critical factor, and its expression level is of paramount importance in regulating the development of leaf serrations [13]. IBR5 and PIN1 are two differently localized proteins in the nucleus and plasma membrane, respectively (Figure 2 and Figure 5). However, the direct link between them remains elusive. Combined with the decreased expression level of PIN1 at the transcriptional and translational level in *ibr5-3* (Figure 5), we propose that IBR5 could act upstream of *PIN1* to activate its expression in leaf serrations via dephosphorylating and activating another transcription factor. Moreover, the phosphorylation of PIN1 is essential for its polar distribution, which determines the auxin transport direction and patterns [39,40]. Hence, this suggests another possible link between IBR5 and PIN1. Because the functionality of IBR5 was achieved by modulating the auxin transport in a PIN1-dependent manner, further efforts should be dedicated to investigating the direct link between these two components.

Previously reported results have demonstrated that the catalytic activity of IBR5 was critical for its function in petal size and root development [26,27]. These results were also supported by our findings that IBR5-GFP_C129S_ failed to rescue the defective leaf serrations of *ibr5-3* (Figure 2). Therefore, the functionality of IBR5 was conferred by its dual-specificity phosphatase activity in the development of leaf serrations, similar to its roles in roots [26]. Hence, the substrate of IBR5 is necessary for a better understanding of its roles in many developmental processes. For instance, MPK12 was identified as one substrate that was efficiently dephosphorylated and inactivated by IBR5, and acted as a negative regulator of auxin signaling in root development [26]. Nevertheless, *MPK12* mutants showed normal leaf serrations as reported [41], which suggested that MPK12 plays no role in IBR5-mediated leaf-serration development. Because of the absence of the consensus sequence recognized by IBR5, it is impossible to evaluate more likely substrates via *in silico* prediction. Based on the subcellular localization, as well as the phenotypes of *IBR5* loss-of-function mutants, we considered that some transcription factors expressed in leaf serrations may be the putative substrates of IBR5, such as *Arabidopsis* CUC2 and CUC3 [9,13]. Overexpression of CUC2 and CUC3 exhibited pronounced and dissected leaf serrations [13], similar to those of *ibr5-3*. CUC2 acts earlier and possibly through limiting growth of the sinus and/or promoting growth of the teeth, whereas CUC3 appears to act later to sustain teeth growth [42]. Moreover, CUC2 activity is required for building up discrete maxima of auxin via a modification of auxin transport [43]. Hence, CUC2 is likely to be a substrate of IBR5 in leaf serration development. Auxin response factors (ARFs) were provided to be phosphorylated and dephosphorylated, and also involved in cell expansion [44,45], which suggests that they may act as substrates of IBR5 in leaf development.

In summary, IBR5 is critical for the development of leaf serrations as phosphatase enzymatic activity is required. Moreover, IBR5 acted through regulating the distribution of auxin maxima at the tips of leaf serrations via modulation of the abundance of PIN1, an observation that provides further support for the importance of auxin dynamics in this process [13,26]. Collectively, this work sheds light on the roles of IBR5 in the development of leaf serrations, which is intriguing and deserves to be studied in the future.

## 4. Materials and Methods

### 4.1. Plant Materials and Growth Conditions

The T-DNA insertion lines, SALK_032185 (*ibr5-2*) and SALK_039359C (*ibr5-3*), were obtained from the European Arabidopsis Stock Center (NASC, http://arabidopsis.info). The upstream and downstream sequences of the inserted sites of *ibr5-2* and *ibr5-3* were amplified via ZP2/ZP12 and ZP3/ZP4, respectively. Then, the PCR product was sequenced and aligned with the sequence of the *IBR5* genomic sequence. The primers are listed in Supplementary Table S1.

All the experiments were conducted in Col-0 background. The plants were grown in a 4:1:1 mix of Fafard 4P:perlite:vermiculite under an 18 h lightness/6 h darkness cycle at 22 °C in the greenhouse of Shanghai Jiao Tong University. For the seedlings’ culture on plates, surface-sterilized *Arabidopsis* seeds were plated on Murashige and Skoog basal medium with vitamins (MS) (Phytotechlab, http://www.phytotechlab.com/). All plates were kept at 4 °C in darkness for 3 days before being transferred to the growth chamber with a 16 h lightness/8 h darkness cycle at 22 °C. Stable *Arabidopsis* transformations were done using the floral-dip method as described [46].

### 4.2. PCR, RT-PCR, qRT-PCR and Vectors Construct

Total leaf RNAs was isolated using TIANGEN RNAprep pure Plant Kit (DP432, http://www.tiangen.com/) according to the manufacturer’s instructions. In total, 1 μg RNA was used to synthesize the first-strand cDNA using the Rever Tra Ace-a-First strand cDNA synthesis kit (TOYOBO, http://www.toyobo.cn/). Primers used for qRT-PCR are as follows: ZP1/ZP2 for the F1/R1 of *IBR5*; ZP3/ZP4 for the F2/R2 of *IBR5*, ZP5/ZP6 for the F3/R3 of *IBR5*, ZP206/ZP207 for *YUC1*, ZP208/ZP209 for *YUC2*, ZP210/ZP211 for *YUC4*, ZP212/ZP213 for *YUC6*, ZP214/ZP215 for *PIN1*, ZP216/ZP217 for *AUX1*, ZP200/ZP210 for GAPDH, ZP202/ZP203 for *ACTIN2*, and ZP204/ZP205 for *TUB2*. Primers for RT-PCR are as follows: ZP5/ZP218 for the F3/GFPR of IBR5-GFP and ZP204/ZP205 for *TUB2*. Detailed information for these primers is listed in Supplementary Table S1.

The qRT-PCR was performed with the Bio-Rad CFX96™ Real-Time System (Bio-Rad, CA, USA) using SYBR^®^ Green Real-time PCR master mix (TOYOBO CO., LTD, Japan). Each 40-µL reaction system contained 20 µL SYBR^®^ Green Realtime PCR master mix, 2 µL cDNA, and 2 µL primers (10 µM). The program was as follows: 95 °C for 1 min and 95 °C for 15 s, 58 °C for 15 s, and 72 °C for 15 s repeated for 46 cycles. Fluorescence data were collected during the 72-°C step and analyzed with the BioRad CFX Manager (Bio-Rad, USA). Total RNA extraction was extracted from the third leaves of 10-day-old plants and with three independent biological replicates. Quantification of relative expression values was performed, and the reference genes were selected as described [47,48].

The vectors mentioned in this work were modified from *pCAMBIA1301*. The functional IBR5-GFP reporter vector driven by its own promotor was constructed using In-Fusion technology (http://www.clontech.com) to insert the IBR5 genomic fragment without a stop codon into *pCAMBIA1301*. The IBR5_C129S_-GFP reporter vector was generated by replacing the 429-bp sequence with a manually synthesized sequence including the point mutation. The pointed sequence was inserted into sequence of IBR5-GFP through two restriction enzyme sites: *XbaI* and *PstI*. The point mutated sequence was synthesized in Sangon Biotech Company (Shanghai, www.sangon.com/). The vectors of IBR5-GFP and IBR5_C129S_-GFP were transformed into *Arabidopsis* via the floral dipping method. For these vectors, more than 15 independent transgenic lines (stable F3 homozygous lines) were obtained and analyzed for each, and only one representative line was exhibited: ZP11/ZP12 for *IBR-GFP*. All primers used in this study are listed in Supplemental Table S1.

### 4.3. Leaf Serration Height and Width, Cell Size, and Cell Number Analysis

Leaf serration height and width analysis was referred to the reported study [9]. The third leaf of 10-day-old plants was fixed in 2.5% glutaraldehyde for 20 min within vacuum. The samples of adaxial cell length measurement were selected from the basal region of the first most proximal serration except the epidermis of the leaf. Images were acquired by the light microscope (E200, Nikon, Japan) and analyzed by the ImageJ (http://rsb.info.nih.gov/ij/).

### 4.4. GUS Staining and Vascular Pattern Analysis

The 10-day-old plants of *ibr5-3* and WT were immersed into GUS solution (50 mM Na_3_PO_4_ (pH 7.0), 50 mM NaH_2_PO_4_, 10 mg/mL X-Gluc and 0.02% (v/v) TritonX-100, 10 mM Na_2_EDTA, 0.5 mM K_3_[Fe(CN)_6_], 0.5 mM K_4_[Fe(CN)_6_]) under dark at 37 °C for 12 h. After that, the samples (stable T3 homozygous lines) were washed with 70% ethanol for 36 h twice until the leaves become transparent. The GUS staining images were taken via a Leica light microscope (M205A) with a CCD camera.

### 4.5. Fluorescence Observation and Analysis

The leaves from10-day-old IBR5-GFP transgenic plants (stable T3 homozygous lines) were captured by epifluorescence using an inverted laser scanning confocal microscope (SP5, Leica, German). DR5-GFP images were captured by an inverted laser scanning confocal microscope (LSM780, Zeiss, German). Next, 15% of Argon laser power was used to excite the sample, the pinhole was set as 86 µm, and the detector ranged from the 505- to the 550-nm band pass filter for GFP and from the 600- to the 650-nm band pass filter for chlorophyll fluorescence, which were placed in front of PMT2. The fluorescence intensity was analyzed via ImageJ (http://rsb.info.nih.gov/ij/) as the subsequent steps: 1) Open the image and draw the regions of interest (ROI) (boxed region containing the PIN1 fluorescence of plasma membrane) via the “rectangle tool”. 2) Select “Analyze–Tools-ROI Manager” and open a dialog box. 3) Select “Analyze–Set Measurements” to select the “Area, Min and max gray value, Integrated density and Mean gray value” to measure. 4) Click on “Measure” in the ROI Manager window. The detailed results were exhibited in a results window and could be saved as Excel files.

## Supplementary Materials

Supplementary materials can be found at https://www.mdpi.com/1422-0067/20/18/4429/s1.

## Abbreviations

AS1	ASYMMETRIC LEAVES1
ARFs	auxin response factors
AUX1	auxin influx carrier
AXR1	AUXIN RESISTANT1
BP	BREVIPEDICELLUS
CUC2	CUP-SHAPED COTYLEDON2
DsPTP1	DUAL-SPECIFICITY PROTEIN TYROSINE PHOSPHATASE 1
IAMT1	Indole-3-Acetic Acid Carboxyl Methyltransferase
IBR5	INDOLE-3-BUTYRIC ACID RESPONSE 5
KNOX	KNOTTED1-like homeobox
MAPKs	Mitogen-activated protein kinases
MKPs	MAPKs phosphatases
PHS1	PROPYZAMIDE HYPERSENSITIVE 1
PIN1	PIN-FORMED 1
WT	wild-type
YUC1	YUCCA1
