# Supplementary material for: IBR5 Regulates Leaf Serrations Development via Modulation of the Expression of PIN1
Source: Int J Mol Sci. 2019 Sep 9;20(18):4429. doi: 10.3390/ijms20184429 (PMC6770195; doi:10.3390/ijms20184429)
Supplement: Supplementary file 1 [file ijms-20-04429-s001.pdf]

International Journal of
Molecular Sciences

MDPI

Article

IBR5 Regulates Leaf Serrations Development via Modulation of the Expression of *PIN1*

Xiuzhen Kong [1,†], Guoqiang Huang [2,†], Yali Xiong [1], Chunyan Zhao [1], Jun Wang[1], Xiaoyun Song[1], Jitender Giri [3] and Kaijing Zuo [1,*]

1 Plant Biotechnology Research Center, School of Agriculture and Life Sciences, Shanghai Jiao Tong University, Shanghai 200240, China; kzxiuzhen@163.com (X.K.); 1732687149@qq.com (Y.X.); zhao13817438337@sjtu.edu.cn (C.Z.); rexue997@126.com (J.W.)songxiaoyun67@163.com (X.S.)
2 State Key Laboratory of Hybrid Rice, School of Life Sciences and Biotechnology, Shanghai Jiao Tong University, Shanghai 200240, China; huang19880901@126.com
3 National Institute of Plant Genome Research, New Delhi 110067, India; jitender@nipgr.ac.in
* Correspondence author: kjzuo@sjtu.edu.cn
† these authors contribute equally to this work (X.K. and G.H.)

Received: 10 July 2019; Accepted: 6 September 2019; Published: 9 September 2019

Supplementary Materials:

This PDF file includes:

Figure S1. Two T-DNA insertion mutants were obtained for *IBR5*.

Figure S2. The sketch for the analysis of leaf-serration height and width.

Figure S3. The height and width analysis of the second, third and fourth serrations at the proximal region of the third leaf.

Figure S4. Cell size and cell number analysis at the most proximal serration.

Figure S5. Only IBR5.1 was detected in the leaves of IBR5-GFP transgenic plants.

Figure S6. Auxin distribution analysis in WT and *ibr5-3*.

Figure S7. The relative expression of auxin biosynthesis genes in WT and *ibr5-3*.

Table S1. Primer used in this work.

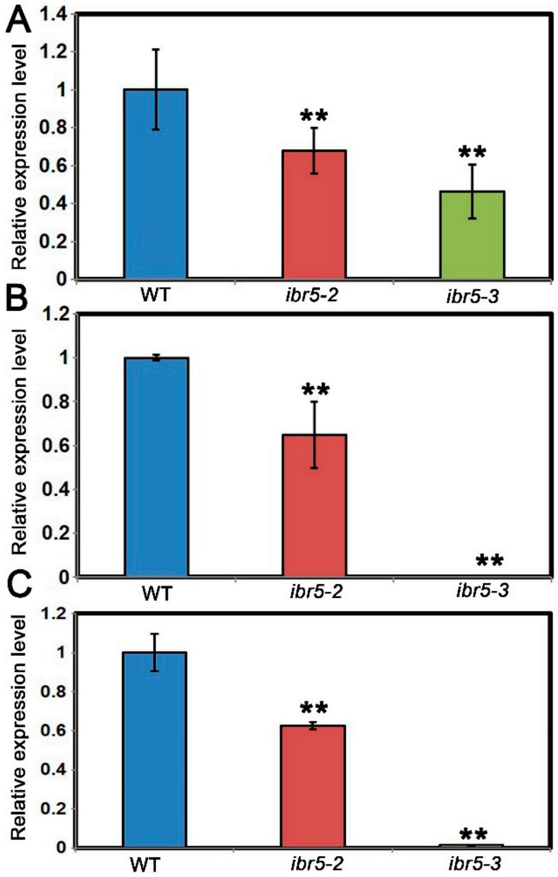

Figure S1. Two T-DNA insertion mutants were obtained for *IBR5*. (**A**) The relative expression level of *IBR5* in WT, *ibr5-2* and *ibr5-3* amplified via 1F and 1R. Error bars are ± SE, n = 3 independent replicates with 3 biological replicates analyzed in each assay. Two asterisks means significant differences ($P < 0.01$ from Student's *t*-test). (**B**) The relative expression level of *IBR5* in WT, *ibr5-2* and *ibr5-3* amplified via 2F and 2R. Error bars are ± SE, n = 3 independent replicates with 3 biological replicates analyzed in each assay. Two asterisks means significant differences ($P < 0.01$ from Student's *t*-test). (**C**) The relative expression level of *IBR5* in WT, *ibr5-2* and *ibr5-3* amplified via 3F and 3R. Error bars are ± SE, n = 3 independent replicates and each with 3 biological replicates analyzed in each assay. Two asterisks means significant differences ($P < 0.01$ from Student's *t*-test).

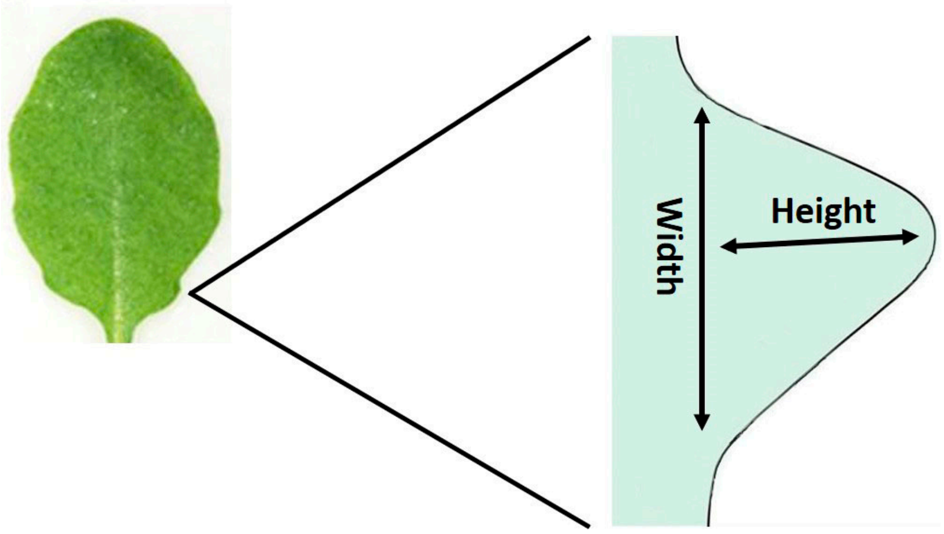

Figure S2. The sketch for the analysis of leaf-serration height and width. The first leaf serration of the third leaf was selected for analysis. The width refers to the maximum length of the leaf serration at the bottom region. The height refers to the vertical length from the tip of leaf serration to its bottom region.

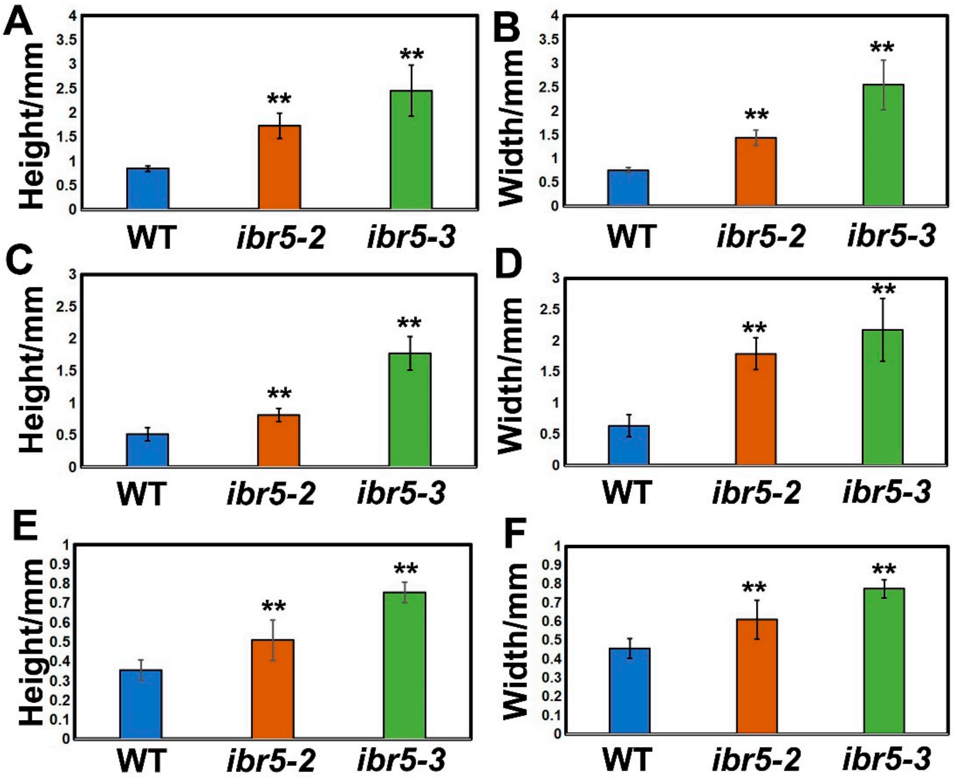

Figure S3. The height and width analysis of the second, third and fourth serrations at the proximal region of the third leaf. (**A**, **B**) The height and width of the second serrations at the proximal region of the third leaf. Error bars are ± SE, n = 3 independent replicates and each with 23 serrations from different leaves analyzed in each assay. Two asterisks means significant differences (*P* < 0.01 from Student's *t*-test). (**C**, **D**) The height and width of the third serrations at the proximal region of the third leaf. Error bars are ± SE, n = 3 independent replicates and each with 25 serrations from different leaves analyzed in each assay. Two asterisks means significant differences (*P* < 0.01 from Student's *t*-test). (**E**, **F**) The height and width of the fourth serrations at the proximal region of the third leaf. Error bars are ± SE, n = 3 independent replicates and each with 21 serrations from different leaves analyzed in each assay. Two asterisks means significant differences (*P* < 0.01 from Student's *t*-test).

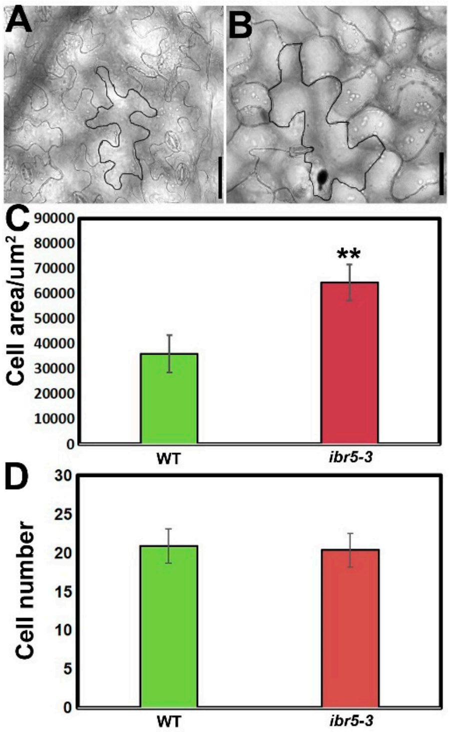

Figure S4. Cell size and cell number analysis at the bottom serration of the third leaf. (**A**) The representative image for the first serration of the third leaf from the 10-day-old WT plant. Bar, 50 µm. (**B**) The representative image for the first serration of the third leaf from the 10-day-old *ibr5-3* plant. Bar, 50 µm. (**C**) Cell area analysis for WT and *ibr5-3*. Error bars are ± SE, n = 3 independent replicates and each with 27 cells of the adaxial domains of most-proximal-serrations from different leaves analyzed in each assay. Two asterisks means significant differences ($P < 0.01$ from Student's *t*-test). (**D**) Cell number analysis for WT and *ibr5-3*. Error bars are ± SE, n = 3 independent replicates and each with 27 the adaxial domains of most-proximal-serrations from different leaves analyzed in each assay.

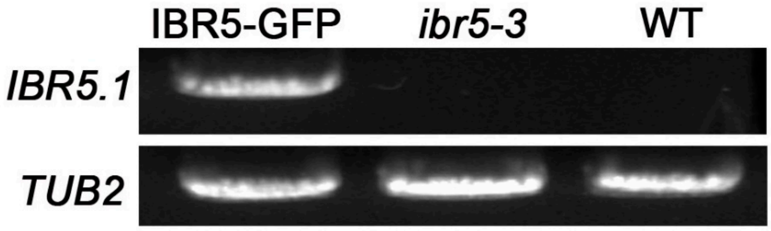

Figure S5. Only IBR5.1 was generated in IBR5-GFP transgenic plants. There are two spliced transcripts (IBR5.1 and IBR5.3) for IBR5. While, IBR5.1 but not IBR5.3 was detected in IBR5-GFP.

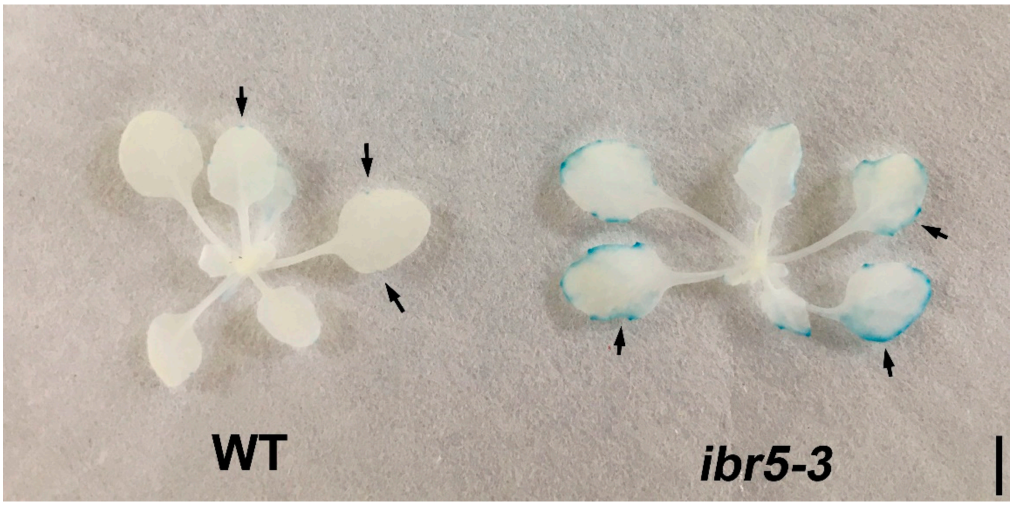

Figure S6. Auxin distribution analysis in WT and *ibr5-3*. The representative images of GUS staining image of 10-day-old DR5-GUS transgenic plant of WT and *ibr5-3*. Bar, 5 mm.

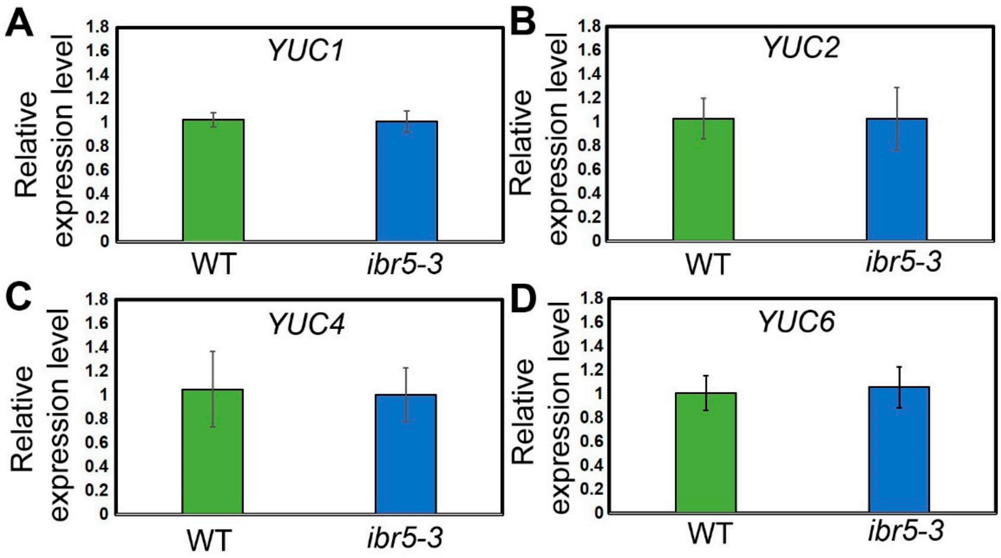

Figure S7. The expression levels of *YUC1*, *YUC2*, *YUC4* and *YUC6* in WT and *ibr5-3*. (**A**) The relative expression level of *YUC1* in WT and *ibr5-3*. Error bars are ± SE, n = 3 independent replicates and each with 3 biological replicates analyzed in each assay. (**B**) The relative expression level of *YUC2* in WT and *ibr5-3*. Error bars are ± SE, n = 3 independent replicates and each with 3 biological replicates analyzed in each assay. (**C**) The relative expression level of *YUC4* in WT and *ibr5-3*. Error bars are ± SE, n = 3 independent replicates and each with 3 biological replicates analyzed in each assay. (**D**) The relative expression level of *YUC6* in WT and *ibr5-3*. Error bars are ± SE, n = 3 independent replicates and each with 3 biological replicates analyzed in each assay.

Table S1.

Primers used in this work

Primer name	Sequence (5' -> 3')
ZP1	AACCCTAATTTCCTCCGTCTGTG
ZP2	ACGGTTCCTATGTGCCAGAATC
ZP3	AGTTACGACAACGCTTCTCGC
ZP4	TGATGAAACGAAAAGGGTGGAGAC
ZP5	TCAGTGGGTTAAACAACGGAGAC
ZP6	TGAGATTGGAAGCATCTTTGTCTGG
ZP11	GATTACGAATTCGAG CATTGTCCGGGTCGGGTTTA
ZP12	CTTGCTCACCATTCGAGAGCCATCCATTGCAATATC ACC
ZP200	TTGGTGACAACAG^GTCAAGCA
ZP201	AAACTTGTCGCTCAATGCAATC
ZP202	CTTGATGTCGGTCTTGTAGG
ZP203	TTCTCCTTGATGTCTCTT
ZP204	GCTGACCACACCTAGCTTTGG
ZP205	AGGGAACCTTAGGCAGCATGT
ZP206	CGGTCGGATTCAATAGCATCTC
ZP207	AAGCGTAGGACTCAAGGTAGG
ZP208	GGATGAGACAATGGAGTATG
ZP209	ATATTTCACCGCTCTTATAGG
ZP210	ACGCATCTGGTCTATGGAATG
ZP211	CGGACTTGTACGCACTGG
ZP212	GGTTGAGTCGGCTGCGTTTG
ZP213	ACATACTCCGTCGTGCCTTCTTC
ZP214	ACAAAACGACGCAGGCTAAG
ZP215	AGCTGGCATTTCAATGTTCC
ZP216	ACTTCTCGACCACTCCAACGC
ZP217	ATCCCAATCACTTTCTCCCAC
ZP218	TGTCGCCCTCGAACTTCAC
